# Application of a Lead Film-Modified CNT/SGC Electrode in the Voltammetric Analysis of Trace Concentrations of Mo(VI)

**DOI:** 10.3390/s26144389

**Published:** 2026-07-10

**Authors:** Malgorzata Grabarczyk, Wieslawa Cwikla-Bundyra, Oliwia Siewierska

**Affiliations:** Department of Analytical Chemistry, Institute of Chemical Sciences, Faculty of Chemistry, Maria Curie-Sklodowska University, 20-031 Lublin, Poland; wieslawa.cwikla-bundyra@mail.umcs.pl (W.C.-B.); oliwiasie@wp.pl (O.S.)

**Keywords:** Mo(VI), carbon nanotubes, spherical glassy carbon, electrochemical sensor, mineral waters

## Abstract

An adsorptive stripping voltammetric method for the determination of ultra trace amounts of Mo(VI) using an electrode based on a mixture of carbon nanotubes and spherical glassy carbon (CNT/SGC) was developed. The electrode was modified by depositing a lead film in situ during each measurement cycle. In a supporting electrolyte containing 0.2 mol/L of acetic buffer pH = 5.3; 0.2 mmol/L of Pb(II); and 0.15 mmol/L of cupferron, the stripping response observed at −0.62 V was proportional to the Mo(VI) concentration within the range of 7 nmol/L to 0.6 µmol/L. The measurement protocol involved a 20 s electrode modification followed by a 30 s adsorption of Mo(VI)–cupferron complexes. The limit of detection was found to be 2.5 nmol/L with a correlation coefficient of 0.997. The method has been applied to the determination of Mo(VI) in mineral water samples.

## 1. Introduction

Molybdenum plays a significant role in everyday life, particularly in relation to many aspects of human health and environmental protection. Molybdenum is a key and essential trace element for both animals and plants, primarily as a component of enzymes that catalyse redox reactions, such as the oxidation of aldehydes and xanthines, and the reduction in nitrates and molecular nitrogen. A molybdenum deficiency in the human diet can lead, for example, to neurological disorders. On the other hand, high concentrations of molybdenum in the body can cause severe irritation of the gastrointestinal tract, heart failure, and disorders of fat and protein metabolism [1,2,3]. Therefore, it is necessary to continuously monitor its concentration in various environmental materials. Molybdenum in the environment originates from both natural geological processes and human activities (anthropogenic sources). It is a trace element that occurs in relatively small quantities in the Earth’s crust, mainly in the form of minerals. Molybdenum is released into soil and water through the weathering of igneous and sedimentary rocks, and it can also enter the atmosphere along with volcanic ash. As for anthropogenic sources of molybdenum in the environment, they are mainly related to the mining and processing of molybdenum ores, and to the fact that it is a by-product of the processing of copper and tungsten ores as well as the combustion of coal and crude oil. Another key factor is the metallurgical and chemical industry, which includes the production of alloy steels, lubricants, catalysts and pigments, as molybdenum is used in their production process. Molybdenum plays an extremely important role in pollution control processes, such as the desulfurization of exhaust gases produced by the combustion of sulfur-containing fossil fuels and the purification of gases from the incineration of municipal and other waste [4,5].

Due to the low concentration of molybdenum in environmental samples, the development of new methods for its quantitative determination poses a challenge. Various analytical methods have been used to determine molybdenum in different matrices, such as spectrophotometry [6], spectrofluorometry [7], inductively coupled plasma mass spectrometry [8], inductively coupled plasma atomic emission spectrometry [9], neutron activation analysis [10], flame atomic absorption spectrometry [11], graphite furnace atomic absorption spectrometry [12], and electrothermal atomic absorption spectrometry [13]. However, these methods, in addition to requiring preliminary concentration and/or separation steps, often necessitate expensive and specialised equipment, which hinders their use in field measurements and small laboratories. Electrochemical methods, on the other hand, can be a good alternative thanks to a number of advantages, such as significantly lower equipment costs, greater portability, and short measurement times, while still enabling the determination of low concentrations at trace levels, as well as good selectivity, precision, and accuracy. The limit of detection for voltammetric methods may be higher than that of competing methods, particularly when compared with ICP-MS; however, given the numerous advantages mentioned above, there is a real need to develop new voltammetric procedures with increasingly better analytical performance, mainly through the use of new electrode materials.

Considering the numerous advantages mentioned above, we employed AdSV (adsorption stripping voltammetry) in our studies to determine trace concentrations of Mo(VI) in mineral waters, as it is one of the most sensitive electrochemical techniques. The most important key aspects in AdSV are the selection of the complexing agent and the electrode material on which the adsorption of electrochemically active complexes formed with the analyte takes place. Mo(VI) forms electrochemically active complexes with many complexing agents. For this reason, numerous examples of their use in AdSV procedures can be found in the literature. These complexing agents include chloranilic acid [14,15,16,17], cupferron [17,18,19,20], alizarin s [21,22,23], oxine [24,25], morine [26], cyclohexane [20], ARS [27], quercetin [28], pyrogallol red [29], pyrocatechol violet [30], and tiron [31]. As can be seen, chloranilic acid and cupferron are among the most commonly used complexing agents. Both are very frequently used in AdSV procedures for the determination of various ions; however, as demonstrated in study [17], in the case of Mo(VI) determination, cupferron provides a lower limit of detection compared to chloranilic acid. Cupferron is a valued analytical reagent used as a complexing agent, mainly due to its unique selective properties in an acidic environment. The cupferron anion binds to metal cations via two oxygen atoms, forming stable five-membered chelate rings, and the complexation reaction proceeds rapidly, which is important in quantitative analysis. Therefore, in our procedure, we used cupferron as a complexing agent to ensure the formation of stable, electrochemically active Mo(VI) complexes that readily adsorb onto various electrode materials.

Another key element in AdSV analytical procedures is the selection of the working electrode material. As with most metal ions, the first AdSV procedures designed for their determination relied on mercury electrodes, primarily in the form of hanging mercury drop electrodes (HMDE) [32], and less frequently in the form of mercury film electrodes (HgFE) [33]. These electrodes provided very good analytical performance, which is why attempts to replace them with less toxic or non-toxic electrode materials have been and still remain a major challenge. Carbon electrodes are currently the primary, environmentally friendly alternative to toxic mercury electrodes in voltammetry, offering a wide range of potentials (particularly in the positive region) and physicochemical stability. Unlike liquid mercury, carbon electrodes are solids with a fixed surface area, which allows them to be reused multiple times after polishing and cleaning. One of the most popular carbon electrode materials is carbon paste, i.e., a mixture of carbon powder and a binder, which offers the advantages of easy preparation, low cost, a porous surface, a wide potential range, low background current, and easy surface renewal. Most often, carbon paste electrodes are chemically modified by adding a suitable modifier. Their properties depend on the properties of the modifying materials, which increase sensitivity to the target depolarizer and, at the same time, the selectivity of the measurements in actual samples [34,35]. For the determination of Mo(VI), the following electrodes of this type have been described in the literature: α-benzoinoxine-modified carbon paste electrode coupled with the catalytic effect on the reduction in chlorate [25], carbon paste electrode modified in situ with cetyl trimethylammonium bromide combined with the reduction of molybdenum(VI)-oxalate [36], a carbon paste electrode modified with sodium dodecyl sulphate combined with the oxidation of the Mo(VI)–morine complex [26], adsorption of the molybdenum–alizarin violet complex on an acetylene black paste electrode followed by its oxidation [37], and a magnetic nickel–zinc ferrite nanocomposite-modified carbon paste electrode [38]. The modification of environmentally friendly carbon electrodes often involves the use of toxic modifiers, a factor that must be taken into account when assessing the environmental profile of such electrodes.

Glassy carbon (GC) is also widely used as an advanced engineering material due to its unique combination of the properties of glass, ceramics, and graphite. Its popularity stems from its excellent chemical resistance, good electrical conductivity, and wide potential window [39]. For Mo(VI) determinations, GC modification is necessary, which is most commonly performed by applying a lead film [19,21,27] or, less frequently, a bismuth film [14]. GC has also been used in the centrifugation–voltammetric determination of molybdenum, which combines the advantages of centrifugation and voltammetry with various carrier reagents, i.e., oxine, pyrogollol red, and cupferron, enabling the formation of a thin film prior to the voltammetric scan [40]. In recent years, the use of carbon-based materials in screen-printed electrodes (SPEs) has become the cornerstone of modern analytical electrochemistry, enabling the production of low-cost, disposable, and versatile sensors. Screen-printing technology is now well-established for the production of reproducible, inexpensive, simple, easy-to-use, portable, single-use sensors. These electrodes are both commercially available and can be prepared in-house by selecting the size, shape of the electrode, and the type of substrate and ink. The modification of SPEs can be performed not only on the surface of the electrode but also in the initial ink. For Mo(VI) determinations, cupferron–tetraphenylborate ion-pair drop-coated modified SPCEs [20], in situ modified SPCEs with a lead film [41], and ex situ modified SPCEs with a bismuth film [42] have been proposed.

In one of the most recent studies, a new indirect voltammetric method for the determination of molybdenum using various polymer-film-modified pencil graphite electrodes was described. Poly-Eriochrome Black T-film-modified pencil graphite electrodes, poly-xylenol orange-modified pencil graphite electrodes, and poly-methyl red-modified pencil graphite electrodes were used for this purpose [43]. Another interesting proposal put forward in recent years for a carbon-based electrochemical sensor is the use of carbon nanomaterials. For this purpose, an electrode based on a mixture of carbon nanotubes and spherical glassy carbon (CNT/SGC) was developed. Both of these advanced carbon nanomaterials are characterised by high electrical conductivity, chemical and mechanical stability, and a large specific surface area, which makes them extremely valuable electrode materials in stripping voltammetry [44,45,46]. The CNT/SGC electrode has previously been successfully used for the determination of metal ions, providing very good analytical performance with a low detection limit, and in this work, it was used for the first time to determine trace amounts of Mo(VI). Because Mo(VI) complexes are not adsorbed directly onto carbon electrodes, such electrodes must be modified for this purpose. In our study, we employed a CNT/GC modification technique involving the in situ formation of a lead film on its surface, as it has been repeatedly demonstrated that electrochemically active complexes of many ions, including Mo(VI), are effectively adsorbed onto metallic lead. Of course, this modification involves the addition of Pb(II) ions to the solution being analysed, from which the film is generated; consequently, the procedure is less environmentally friendly. However, the concentrations involved are usually negligible below mmol/L and are added to a small volume of the solution being analysed, often ≤10 mL.

## 2. Materials and Methods

### 2.1. Apparatus

All voltammetric measurements were undertaken at room temperature with a μAutolab analyser (Utrecht, The Netherlands) coupled to an electrochemical stand made by MTM Cracow, Poland. A three-electrode electrochemical cell arrangement was used with the multiwall carbon nanotubes/spherical glassy carbon electrode (CNTs/SGCE) as the working electrode, Ag/AgCl (saturated NaCl) as the reference electrode, and a platinum wire as the auxiliary electrode. The ultrasonic bath test was carried out using the Sonic-3 (Polsonic, Warsaw, Poland).

### 2.2. Reagents and Chemical

Solutions were prepared in water obtained from a Millipore Elix Advantage 15 (Molsheim, France) using analytical grade reagents, unless otherwise stated. The solutions of 1 mol/L of the acetate buffers were prepared from Suprapur CH_3_COOH and NaOH obtained from Merck. The solution of cupferron at concentrations of 10 mmol/L was received by dissolving 0.031 g of the reagent in deionized water in a 20 mL small bottle and was stored in a refrigerator at a temperature of 6 °C. The working solutions of Ag(I), Al(III), Bi(III), Co(II), Cr(III), Cu(II), Fe(III), Ge(IV), Hg(II), Mg(II), Mn(II), Mo(VI), Ni(II), Pb(II), Sn(II), V(V) and Zn(II) were prepared by appropriate dilution of 1 g/L stock standard solutions (Merck; Darmstadt, Germany). Multiwall carbon nanotubes, O.D. × I.D. × L 10 nm ± 1 nm × 4.5 nm ± 0.5 nm × 3~6 μm with ≥98% carbon basis (Sigma-Aldrich; St. Louis, MO, USA), were used without functionalization. Spherical glassy carbon powder size 0.4–12 µm (HTW Hochtemperatur-Werkstoffe GmbH, Thierhaupten, Germany) was used.

### 2.3. Working Electrode Preparation and Modification

**Figure 1:**
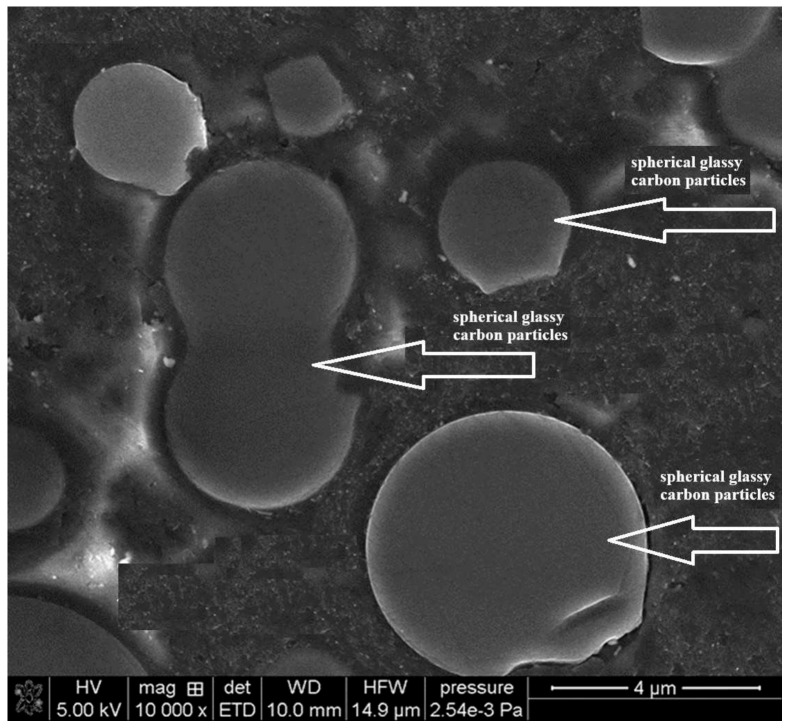
A real image of the solid CNT/SGC electrode taken using a scanning electron microscope (Quanta 3D FEG(FEI): magnification 10,000×.

A working electrode of our own production was prepared in our laboratory using carbon nanomaterials such as carbon nanotubes and spherical glassy carbon. Carbon nanotubes are technically classified as 1D (one-dimensional) nanomaterials because their length is significantly greater than their diameter (which is on the nanometre scale). However, in the context of their application and structure in materials engineering, carbon nanotubes often intertwine to form complex, three-dimensional networks. Spherical glassy carbon is a 3D material; it is a special, porous form of glassy carbon characterised by spherical pore shapes and a unique microparticle structure. The preparation of the CNT/SGC electrode involved first mixing carbon nanotubes with epoxy resin (in a 1:25 ratio), and then mixing the resulting mixture with spherical glassy carbon (in a 2:1 ratio). The final mixture must be centrifuged to remove air bubbles. These proportions were selected on the basis of experiments carried out. In the case of the mass ratio of CNTs to epoxy resin, the amount of CNTs cannot be too small, as this results in poorer conductivity; conversely, if the amount of CNTs is too high, the mixture becomes too dense and air bubbles cannot be removed during centrifugation. As for the amount of SGC, these particles must not be too numerous, as they would then be arranged too close together, causing the electrode to lose its microelectrode properties. The resulting paste was injected under pressure into a 2 mm diameter hole drilled in PEEK. To ensure full hardening, the electrode was placed in a drying oven at a temperature of 105 °C for 48 h. After self-curing, we obtained a functional electrode based on an epoxy resin serving as a matrix in which conductive nanoparticles—namely carbon nanotubes and spherical glassy carbon particles acting as microelectrodes—are embedded. The morphology of the CNT/SGC electrode was examined using a scanning electron microscope (Quanta 3D FEG (FEI, Thermo Fisher Scientific, Waltham, MA, USA)) and was analysed at an accelerating potential of 5 kV using an ETD detector under high-vacuum conditions; the findings are shown in Figure 1. The image illustrates spherical vitreous carbon particles surrounded by numerous nanotubes embedded in resin (little white dots).

Finally, the CNT/SGC electrode was polished on sandpaper, first with coarse grit (P120) and then with fine grit (P2000). Next, to remove any remaining polishing material, the electrode was rinsed with water and placed in an ultrasonic bath for 30 s. The electrode was then ready for measurements and ensured stable and repeatable results. Additionally, on each measurement day, the electrode was polished once with a 0.3 µm thick aluminium oxide slurry on a Buehler polishing block and placed in an ultrasonic bath for 30 s. In each measurement cycle, the electrode was electrochemically modified in situ by depositing a lead film from the sample solution, to which 0.2 mmol/L Pb(II) was added, using a potential of −1.1 V for 20 s.

### 2.4. Typical AdSV Measurement

A 10 mL base solution contained: a synthetic Mo(VI) solution used to optimise the procedure or the sample being analysed; 0.2 mol/L acetic buffer (pH = 5.3) as a supporting electrolyte; 0.2 mmol/L Pb(II) as a modifier; and 0.15 mmol/L of cupferron as a Mo(VI) complexing agent. Each voltammetric measurement began with electrode modification as described above, and then the potential was shifted to −0.6 V for 30 s; during this time, adsorption of Mo(VI)–cupferron complexes occurred on the previously formed lead film, and after a 5 s equilibration time, a voltammogram was recorded as the potential was varied from −0.55 V to −1.0 V, obtaining a peak at a potential of −0.62 V resulting from the reduction in Mo(VI) to Mo(V). The voltammogram was recorded using the pulse-differential technique with a modulation time of 0.003 s, an interval time of 0.1 s, a step potential of 0.015 V, and a modulation potential of 0.1 V. All measurements were conducted at room temperature.

## 3. Results

### 3.1. Evaluation of Modification Step

Electrode modification is most often aimed at improving their sensitivity and selectivity and can be carried out using various methods, such as physical, chemical, or electrochemical methods based on the reduction in precursors from solution. In our procedure, we used electrochemical modification, with Pb(II) ions serving as precursors, from which a lead film was generated on the CNT/SGCE as a result of reduction. Lead on the working electrode creates active catalytic centres, resulting in a current signal with a high signal-to-noise ratio. Furthermore, coating the electrode with lead alters the kinetics of the electron transfer process. On the surface modified with a lead film, the activation energy of the redox process for the complexes under investigation is reduced, allowing for a faster and more efficient exchange of electrons between the analyte and the electrode.

**Figure 2:**
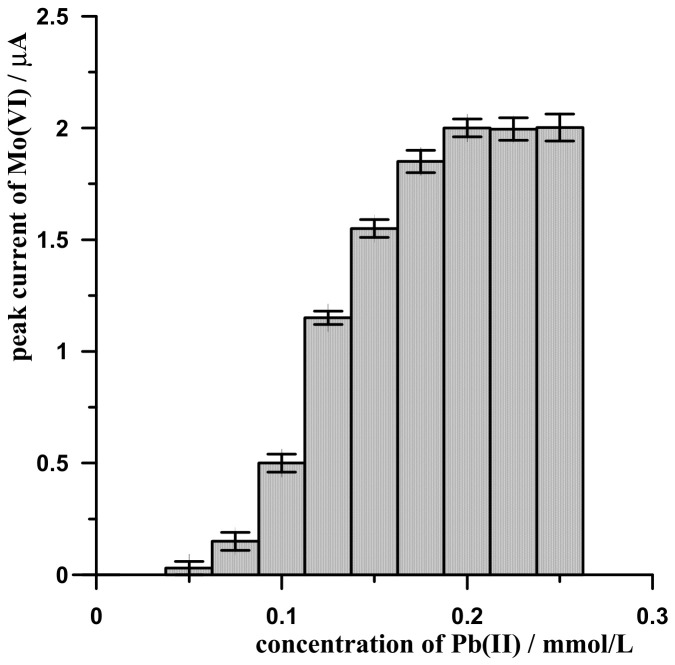
The dependence of Pb(II) concentration on analytical signals of 0.1 µmol/L Mo(VI).

The key parameters determining the quality of the lead film are the concentration of the precursor, i.e., Pb(II) ions in the solution, as well as the potential and duration of the modification; therefore, we evaluated these parameters. The evaluation was carried out by examining the magnitude and shape of the Mo(VI) analytical signal recorded while varying the tested parameter within a specific range, while keeping the remaining parameters constant. The effect of Pb(II) concentration in the solution was analysed using constant measurement conditions, i.e., 0.1 µmol/L Mo(VI); 0.2 mol/L of acetic buffer at pH 5.3; 0.15 mmol/L of cupferron; −1.1 V for 20 s as the potential and time of modification; and −0.6 V for 30 s as the potential and time of adsorption of Mo(VI)–cupferron complexes. It was found that, in the absence of Pb(II) ions in the solution, no molybdenum peak was observed, confirming that Mo(VI) complexes do not adsorb directly onto the CNT/SGCE surface and that its modification is necessary. As the concentration of Pb(II) in the solution increased from 0.05 to 0.2 mmol/L, the molybdenum peak gradually rose until it reached a constant value. Further increases in the concentration of Pb(II) in the solution no longer caused the molybdenum peak to rise, as shown in Figure 2.

**Figure 3:**
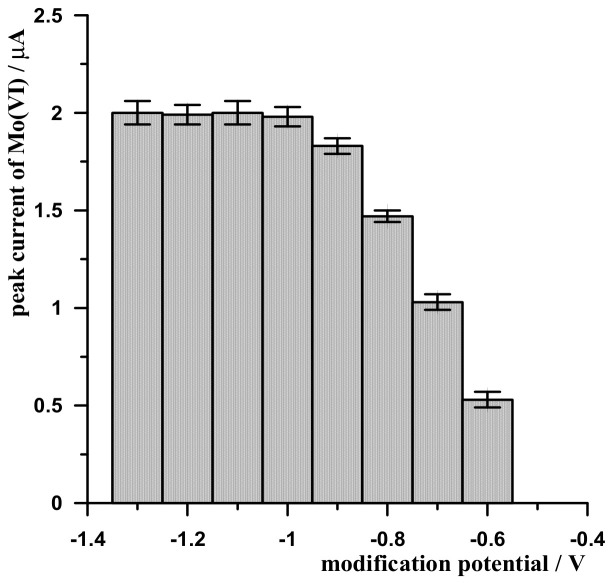
The dependence of modification potential on analytical signals of 0.1 µmol/L Mo(VI).

The next parameter evaluated was the modification potential, which was varied in the range from −1.3 V to −0.6 V while the remaining parameters were kept constant, i.e., 0.1 µmol/L Mo(VI); 0.2 mol/L of acetic buffer, pH = 5.3; 0.2 mmol/L of Pb(II); 0.15 mmol/L of cupferron; 20 s of modification time; and −0.6 V for 30 s as the potential and time for adsorption of Mo(VI)–cupferron complexes. The results obtained are presented in Figure 3.

It was observed that, within the modification potential range of −1.3 to −1.0 V, the molybdenum peak reached its highest constant value; at lower modification potential values, the analytical signal for molybdenum decreased. Under identical conditions, the effect of varying the modification time on the molybdenum signal was investigated using a constant value of −1.1 V. In this case, it was found that the molybdenum peak increased as the modification time was extended up to 20 s; further prolongation of the time did not result in any further increase in the molybdenum analytical signal. Taking into account the main parameters affecting the structure of the lead film—namely, the concentration of Pb(II) ions in the solution, and the potential and time of film formation—the following were selected as the most optimal for obtaining the highest Mo(VI) signal: a Pb(II) concentration of 0.2 mmol/L, and a potential and time of film formation of −1.1 V and 20 s, respectively.

### 3.2. Evaluation of Supporting Electrolyte and Complexing Agent

**Figure 4:**
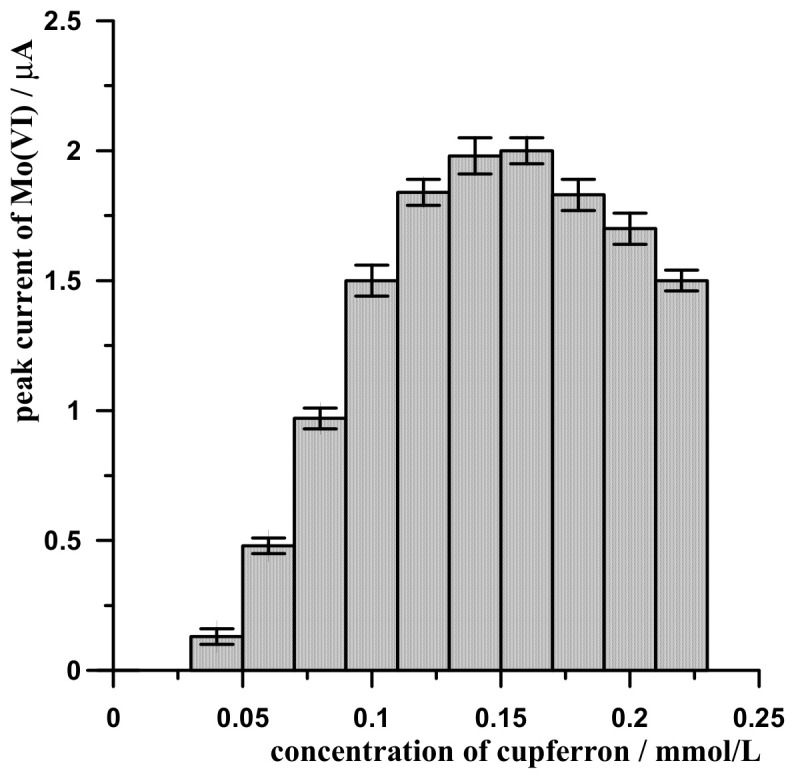
The dependence of cupferron concentration on analytical signals of 0.1 µmol/L Mo(VI).

Based on the literature, a 0.2 mol/L acetate buffer was selected as the base electrolyte, and this study focused on determining its pH. To this end, a series of buffer solutions were prepared in the pH range of 4 to 6 with an accuracy of ±0.05, and their effect on the molybdenum peak height was examined. As observed, the highest signal was obtained for pH values in the range of 5.0–5.7, and on this basis, an acetate buffer with a pH of 5.3 was used for further studies. The optimal concentration of cupferron as a complexing agent was determined by varying its concentration and analysing how it affects the height of the molybdenum peak. The measurements were performed using a solution with the following constant parameters: 0.1 µmol/L Mo(VI); 0.2 mol/L of acetic buffer, pH = 5.3; 0.2 mmol/L of Pb(II); −1.1 V for 20 s as the potential and time of modification; and −0.6 V for 30 s as the potential and time of adsorption of Mo(VI)–cupferron complexes. The molybdenum peak was only visible at a cupferron concentration of 0.04 mmol/L and increased as its concentration rose to 0.15 mmol/L, after which it began to gradually decrease (Figure 4). As can be seen, when the concentration of cupferron is too high, the molybdenum signal decreases; this may be due to competitive adsorption of molybdenum onto the electrode, thereby blocking the surface where the adsorption of Mo(VI)–cupferron complexes takes place. In summary, 0.2 mmol/L acetic acid at pH 5.3 and 0.15 mmol/L cupferron were selected as the most optimal conditions, serving as the supporting electrolyte and complexing agent, respectively.

### 3.3. Evaluation of Mo(VI)–Cupferron Adsorption Step

**Figure 5:**
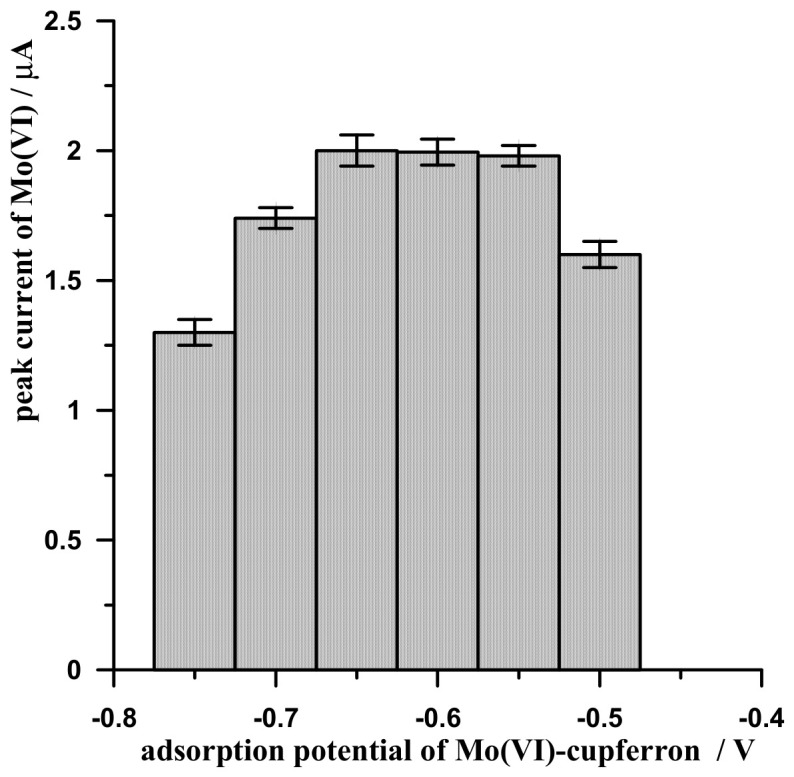
The dependence of adsorption potential of Mo(VI)–cupferron on analytical signals of 0.1 µmol/L Mo(VI).

The adsorption of the Mo(VI)–cupferron complex onto the CNT/SGCE surface using the AdSV technique is a key step in the concentration of the analyte, occurring at a constant potential. This potential was selected by analysing the peak intensity of molybdenum recorded from a solution containing 0.1 µmol/L Mo(VI); 0.2 mol/L of acetic buffer (pH = 5.3); 0.2 mmol/L of Pb(II); and 0.15 mmol/L of cupferron. The potential and modification time were −1.1 V for 20 s, while the complex adsorption potential varied in the range from −0.5 to −0.75 V in 0.05 V increments for a fixed adsorption time of 30 s. It was found that the molybdenum peak reached its maximum value in the potential range from −0.55 V to −0.65 V; at lower or higher potential values, the peak current decreased, which is why an adsorption potential of −0.6 V is generally recommended. Detailed results of these studies are demonstrated in Figure 5. Using an adsorption potential of −0.6 V, the change in the molybdenum peak intensity was examined in a similar manner by varying the adsorption time in the range from 10 to 40 s at 5 s intervals. It was observed that the molybdenum peak increased with increasing time up to 30 s; as the adsorption time increases, the peak current no longer rises and remains at the same level. Given that the most efficient adsorption of Mo(VI)–cupferron complexes on the lead film-modified CNT/GC electrode occurs at a potential of −0.6 V, and that extending the adsorption time beyond 30 s no longer results in an increase in the peak, these parameters were selected as the most optimal.

### 3.4. Evaluation of Differential Pulse Parameters

Optimisation of the following instrumental parameters of differential pulse (DP) technique was performed: step potential [V], modulation amplitude [V], modulation time [s] and interval time [s]. The step potential was measured in the range from 0.005 to 0. 03 V in 0.005 V increments. It was observed that, as the molybdenum peak potential increased, the peak shape deteriorated at higher potential values; therefore, as a compromise between peak amplitude and shape, a step potential of 0.015 V is recommended.

In the case of amplitude modulation, its value was varied in the range from 0.04 V to 0.1 V, with a step size of 0.02 V. In this case as well, the peak intensity increased with increasing potential; therefore, a potential of 0.1 V was selected as the most optimal.

The modulation time was varied between 0.003 and 0.008 s, and it was observed that the peak decreased as the modulation time increased; therefore, a modulation time of 0.003 s was used as standard.

The interval time was varied in the range from 0.1 s to 0.4 s, with a frequency of 0.05 s. In this case as well, the molybdenum peak decreased as the interval time increased; therefore, a value of 0.1 s was used as the standard.

In concluding, it was found that the parameters of the DP technique such as step potential [V], modulation amplitude [V], modulation time [s] and interval time [s] for which the highest molybdenum signal is obtained are 0.015 V, 0.1 V, 0.003 s and 0.1 s respectively, and these are recommended as the most optimal.

### 3.5. Analytical Characteristics of the Procedure

**Figure 6:**
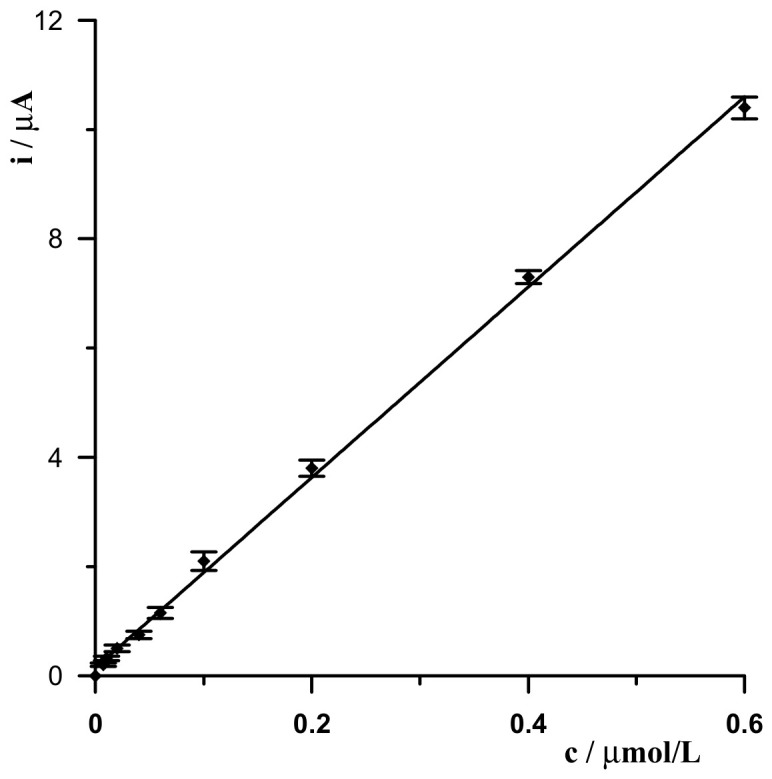
The calibration graph performed using a lead film-modified CNT/SGC electrode for the following solution: 0.2 mol/L of acetic buffer (pH = 5.3), 0.2 mmol/L of Pb(II), 0.15 mmol/L of cupferron, Mo(VI) in the concentration range from 7 nmol/L to 0.6 µmol/L.

The study was conducted using the DP-AdSV technique and optimal conditions determined on the basis of previous experiments. A solution containing 0.2 mol/L of acetic buffer (pH = 5.3), 0.2 mmol/L of Pb(II) and 0.15 mmol/L of cupferron, and increasing concentrations of Mo(VI), was added to it; a voltammogram was recorded after each addition. Based on the results obtained, a relationship between the peak current and Mo(VI) concentration was established, and a calibration curve was determined, which proved to be linear in the concentration range from 7 nmol/L to 0.6 µmol/L and described by the equation y = 17.42x + 0.15, where y and x are the peak current (μA) and concentration of Mo(VI) (µmol/L), respectively (Figure 6). The coefficient of determination (R^2^) of the linear regression equation was 0.977. The detection limit estimated from three times the standard deviation at low Mo(VI) concentration was 2.5 nmol/L. The optimal electrochemical conditions for Mo(VI) determination used to ensure maximum sensitivity, accuracy, and efficiency in the experimental measurements are summarised in Table 1 and were applied in the studies. Table 2 shows a comparison of the lead film-modified CNT/GC electrode with other modified carbon electrodes used in the Mo(VI) analysis.

The repeatability of the measurements was assessed based on six consecutive measurements using different solutions at a constant Mo(VI) concentration of 0.1 µmol/L, expressed as the relative standard deviation (RSD), and was 3.3%. Reproducibility was assessed based on measurements taken over five consecutive days for the same Mo(VI) concentration as RSD and was 3.8%.

### 3.6. Investigating the Interference of Other Cations

To investigate the effect of different cations on the voltammetric signal of Mo(VI), additional experiments were conducted using the DP-AdSV method combined with modified CNT/SGC electrode. These experiments were carried out in the presence of different concentrations of selected possible interfering cations. The voltammetric signal of the electrode was first recorded in the presence of a 0.2 µmol/L solution of Mo(VI). Then, the concentration of the tested cations in the analyte solution was increased in 10-, 50- and 100-fold excess relative to Mo(VI) and for each case a voltammetric signal was recorded. Finally, the recorded signals were compared with the voltammetric signal obtained in the absence of these species. If the difference between this signal and the main signal is less than 5%, it is possible to selectively determine Mo(VI) in the presence of these species without reducing sensitivity. Analysis of the results showed that a 100-fold excess of Ag(I), Bi(III), Co(II), Cr(III), Cu(II), Fe(III), Ge(IV), Mg(II), Mn(II), Ni(II), Sn(II), and Zn(II) ions does not interfere with Mo(VI) determinations. A 50-fold excess of Al(III), Hg(II), and V(V) causes a reduction in the molybdenum peak by 30%, 50%, and 80%, respectively. In the case of V(V), the interference results from the fact that it also forms electrochemically active complexes with cupferron, which are also adsorbed onto the lead film, giving a reduction peak at a potential close to that of the Mo (VI)–cupferron reduction peak; consequently, at higher concentrations of V(V) in the solution, it causes a reduction in or even disappearance of the Mo(VI) peak. However, considering that in natural waters the molybdenum content is estimated at an average of 10 µg/L, whilst the vanadium content, depending on the type of water, ranges from 0.2 μg/L to 100 μg/ L in freshwater (depending on the presence of effluents and leachates from anthropogenic and/or natural sources entering the water) and 3 μg/L in seawater, the proposed procedure can be used for the determination of Mo(VI) in natural waters [47,48].

### 3.7. Analysis of Mineral Waters for Their Content of Mo(VI)

Depending on the source, mineral water can contain as many as 70 different minerals. The most common are macrominerals such as calcium, magnesium, potassium and sodium, as well as trace elements such as iron, manganese, zinc, copper, chromium, iodine, and fluoride, but mineral waters also contain many other microminerals present in much smaller amounts, including molybdenum. Therefore, drinking mineral water not only ensures proper hydration of the body but also supplies it with the elements necessary for humans to function properly, e.g., molybdenum.

**Figure 7:**
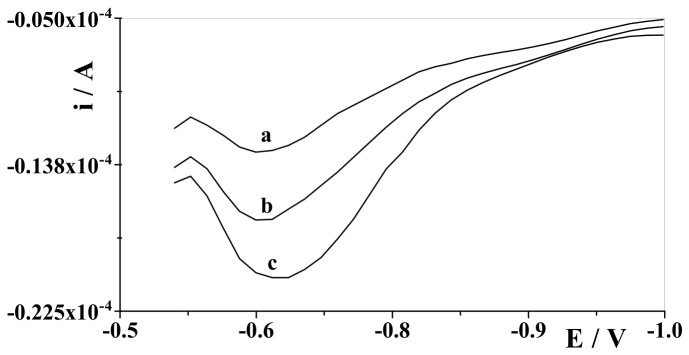
Differential pulse voltammograms obtained in the course of the Mo(VI) determination in mineral water sample: (a) mineral water spiked with 200 nmol/L Mo(VI) the day before diluted two times; (b) as (a) + 100 nmol/L Mo(VI); (c) as (a) + 200 nmol/L Mo(VI).

The practical objective of our study was to use the developed procedure to analyse mineral waters for their Mo(VI) content. Two mineral waters were selected for the study, but none of them contained Mo(VI) above the detection limit of the developed procedure. Therefore, recovery studies were carried out by adding known concentrations of Mo(VI) to the tested mineral waters, and the voltammograms were recorded following the described procedure. The mineral waters were spiked with appropriate concentrations of Mo(VI) and analysed the following day using the standard addition method. The results summarised in Table 3 are satisfactory, indicating the method’s capability for determining molybdenum in mineral water samples. Examples of voltammograms recorded during the analysis of mineral water are presented in Figure 7.

## 4. Conclusions

This work demonstrates that the CNT/SGC electrode is a promising analytical tool, which can be used as a simple sensor able to detect trace amounts of Mo(VI) by differential pulse voltammetry. The electrode’s high performance was achieved through the use of carbon nanomaterials, which, after modification with a lead film, enabled a very low detection limit of 2.5 nmol/L, which was much lower than the maximum value of 70 µg/L Mo(VI) in drinking water permitted by the WHO (corresponding to approximately 730 nmol/L). To the best of our knowledge, this is the first Mo(VI) determination procedure to utilise an electrochemical sensor based on a mixture of carbon nanotubes and spherical glassy carbon. The adsorption voltammetric protocol was based on the efficient adsorption of electrochemically active Mo(VI) complexes with cupferron, and the total measurement time was less than one minute. The sensitive and stable sensor was successfully applied for the determination of Mo(VI) in mineral waters.

## Figures and Tables

**Table 1:** The optimal electrochemical conditions for Mo(VI) determination.

Parameter	Value	Unit
modification potential	−1.1	V
modification time	20	s
adsorption potential	−0.6	V
adsorption time	30	s
initial potential	−0.55	V
end potential	−1.0	V
step potential	0.015	V
amplitude modulation	0.1	V
modulation time	0.003	s
interval time	0.1	s

**Table 2:** The comparison of the lead film-modified CNT/SGC electrode with other modified carbon electrodes used in the Mo(VI) analysis.

Type of Modified Carbon Electrode	Technique	Complexing Agent	Linearity Range [mol/L]	Detection Limit [mol/L]	Time [s]	Ref.
lead film-modified glassy carbon	AdSV	Alizarin S	2 × 10^−9^–5 × 10^−8^	9 × 10^−10^	60	[21]
bismuth film-modified glassy carbon	AdSV	chloranilic acid	5.2 × 10^−8^–5.2 × 10^−7^	2.1 × 10^−9^	600	[14]
α-benzoinoxime modified carbon paste electrode	CV	-	1 × 10^−7^–8 × 10^−5^	2 × 10^−9^	60	[25]
modified screen-printed carbon electrode	AdSV	cyclohexanone	5.2 × 10^−8^–2.1 × 10^−6^	2.6 × 10^−8^	720	[20]
modified carbon paste electrode	AdSV	morin	8 × 10^−10^–6 × 10^−8^	4 × 10^−10^	120	[26]
lead film-modified glassy carbon	AdSV	cupferron	3 × 10^−8^–1 × 10^−6^	9.0 × 10^−9^	50	[19]
cetyltrimethylammonium bromide modified carbon paste	ASV	oxalate	4.2 × 10^−9^–4.2 × 10^−6^	4.2 × 10^−9^	600	[36]
sodium dodecyl sulfate modified carbon paste	ASV	morin	8 × 10^−10^–6 × 10^−8^	4 × 10^−10^	120	[26]
acetylene black paste electrode	AdSV	alizarin violet	6 × 10^−9^–1 × 10^−5^	2 × 10^−9^	120	[37]
lead film-modified glassy carbon	AdSV	ARS	2.8 × 10^−8^–1.25 × 10^−6^	8.2 × 10^−9^	60	[27]
magnetic nickel–zinc ferrite nanocomposite-modified carbon paste	ASV	-	5.2 × 10^−8^–1.04 × 10^−5^	3.1 × 10^−8^	90	[38]
lead film-modified CNT/SGC	AdSV	cupferron	7 × 10^−9^–6 × 10^−6^	2.5 × 10^−9^	50	this work

**Table 3:** The results of Mo(VI) determination in mineral water samples spiked with different concentrations of Mo(VI) (*n* = 6).

Mineral Water Sample	Concentration of Mo(VI) [nmol L^−1^]		Recovery [%]
	Spiked	Found	
Water 1	200	186 ± 7	93.0
	400	391 ± 15	97.7
Water 2	200	185 ± 8	92.5
	400	385 ± 17	96.2

## Data Availability

The original contributions presented in the study are included in the article; further inquiries can be directed to the corresponding author.
